# Provider views on vasectomy: cultural, gender, and political elements of Men's decisions to seek publicly funded services

**DOI:** 10.3389/frph.2024.1386244

**Published:** 2024-12-18

**Authors:** Michelle Teti, Denise Raybon, Stephanie Spitz, Shelby Webb, Jacki Witt, Kristin Metcalf-Wilson

**Affiliations:** ^1^ Honors College, University of Missouri, Columbia, MO, United States; ^2^ Collaborative Center to Advance Health Services, University of Missouri Kansas City, Kanas City, MO, United States

**Keywords:** men & masculinity, men's health, reproductive health, Roe v Wade, vasectomy, qualitative research methods

## Abstract

**Introduction:**

Evidence suggests a new demand for vasectomies following the Supreme Court's Dobbs v. Jackson Women's Health Organization [597 U.S. 215 (2022), (Dobbs)]. Vasectomies are safer and more cost-effective than tubal sterilization. Understanding how to support men's use of this procedure is important to improving sexual and reproductive health and wellbeing (SRHW). This study is an exploration of health care providers' perspectives on the gender, cultural and political influences on vasectomies at Title X-funded clinics across all regions of the US.

**Methods:**

Providers or health services staff (*N* = 21) at Title X-funded settings currently offering vasectomy services in their own clinics or via referral were recruited using list serve and email outreach. Participants took part in one-on-one or small group interviews about vasectomy procedures, patient experiences, and trends. A thematic analysis of interview transcripts through an iterative process of reviewing, note-taking, and discussing data assessed provider views on patient cultural, gender, and political vasectomy influences.

**Results:**

Qualitative interviews yielded four themes related to participant access to services, including income challenges, language barriers, medical distrust, and societal gender roles that stressed women's responsibility for contraception. Two additional themes focused on men's fear of losing reproductive health options and desire to “step up” to do their part to prevent unplanned pregnancies.

**Discussion:**

Interviewees stressed that vasectomy was for everyone but identified sub-groups of men who still faced logistical and social access challenges to the procedure. Providers also believed that men were concerned over reproductive justice in the US and wanted to do their part to help prevent unplanned pregnancies. They thought that the Dobbs decision may mark a turning point in reproductive care that could ultimately better public health initiatives and overall SRHW by including men in the conversation. Vasectomy education, marketing—along with policy changes that ease access, can support this goal.

## Introduction

Sexual and reproductive health and wellbeing (SRHW) is an important component of men and women's overall health. SRHW means fewer sexually transmitted infections, less unintended pregnancies, and decreased subsequent poor family health outcomes like mental distress and violence (1). Having the freedom to make sexual and reproductive health decisions and seek preferred methods of contraception also comprise SRHW (2). This includes access to a full range of SRH care for all, across age, gender, sexuality, race/ethnicity, and income levels (2). These reproductive justice benchmarks will lead to better overall health outcomes for families and communities. To that end, recent studies suggest that expanding contraceptive access to better include frequently desired but lesser used methods like vasectomy (3), and specifically focusing more on developing men's reproductive agency and shared responsibility (4), will improve SRHW.

Most of the research and medical options for contraception focus on the female reproductive system (1). Contraception options for men^**1**^ remain limited, although women want holistic SRH options and most men do believe men *and* women should share in contraception responsibility and want to have input on fertility and reproductive outcomes (5, 6). Vasectomy is one of the few contraceptive methods available for men (7). It is safer and more cost-effective than tubal ligation. Yet, rates of tubal sterilization are consistently higher. For instance, less than 6% of current contraceptive users in the United States rely on vasectomy, compared with 18% opting for tubal sterilization (8). Vasectomy rates are even lower among racial/ethnic minority men (9).

Recent evidence suggests a renewed interest in and demand for vasectomies following the Supreme Court's Dobbs v. Jackson Women's Health Organization [597 U.S. 215 (2022)], overturning Roe v Wade [Roe v. Wade, 410 U.S. 113 (1973)]. There was a peak in information searching for vasectomy on google two weeks following the Dobbs decision when compared to previous trends (10). When the Supreme Court decision was leaked similar increases were noted (10). In addition, female medical students indicate more interest in learning about vasectomies than they did a year prior to the decision (11). Raevti et al., 2023 compared the number of men presenting for vasectomies pre- and post-Dobbs decision in a high-volume medical institution. Their findings show increased vasectomy procedural volumes (12). Likewise, in the seven months after the overturning of Roe, 0.233% of U.S. vasectomy-naive men receiving any outpatient clinical evaluation underwent vasectomy, representing a 20% increase in vasectomy incidence from the 7 months prior (13). Likewise, Bole found that vasectomy consultations increased post-Dobbs among younger men, especially those under 30, as well as child-free men, suggesting that men are invested in maintaining reproductive autonomy for themselves and their partners (12).

The Dobbs decision may mark a turning point in reproductive health history (13). Men may be more inclined and motivated to learn about male options for preventing pregnancy, which can ultimately greatly improve SRHW overall (13, 14). In the long run the decision may trigger increased interest in contraceptive practices that in turn will influence better public health initiatives (15).

Publicly funded sexual reproductive health care providers such as health departments, federally qualified health centers, Planned Parenthood clinics, and community health centers provide safety net services for low-income clients. Many of these organizations receive funding from the federal Title X Family Planning program, which means they are required to offer a broad range of contraceptive methods on-site or by referral. The Affordable Care Act (ACA) requires coverage for tubal sterilization under the ACA Medicaid expansion but does not require coverage for vasectomy. However, in most states vasectomy services are covered through Medicaid and/or Medicaid Family Planning Expansion funds (extension of Medicaid eligibility for family planning services) (16). There is limited published information on vasectomies provided by publicly funded agencies, which can improve SRHW outcomes for marginalized communities (16).

There are many barriers for men accessing vasectomy services, especially men from historically vulnerable groups like low-income men and racial/ethnic minority men. In a study by White et al., only half of the racially and ethnically diverse survey respondents demonstrated accurate knowledge of vasectomy, particularly as it pertains to the perceived impact on sexual functioning (17). Similarly, White et al. also found that men of color and low-income respondents demonstrated less knowledge than white men and those with higher incomes. This suggests that low-income men and men of color lack access to information about the procedure, possibly via less contact or access to health care overall. The cost of vasectomy is seldom transparent, which may further deter men with less resources (18).

Federal regulations regarding vasectomy procedures require a client seeking a federally funded vasectomy to complete a standardized consent form at least 30 days prior to the procedure. While the original intention of this regulation was protective against coercion or other abuses, this restriction has become a barrier to access for clients seeking effective, permanent contraception—which may be worse for men with low incomes or less secure employment, who cannot easily manage multiple steps and appointments (19–21).

Given increased interest and discussion in men's role in contraception, however, particularly post the Dobb's decision, more information is needed about the factors that influence vasectomy, to better design and let all families know about and access the procedure. In this study, we explored Title X provider perspectives on gender, cultural and political influences in vasectomy services to understand how to improve vasectomy services post-Dobbs.

## Methods

### Participants

The research team recruited participants for this study using list serves and emails sent to different Title X grantees identified through the HHS Office of Population Affairs and the Clinical Training Center for Sexual and Reproductive Health. These list serves reached around 10,000 different providers. Providers or health services staff from all U.S. states were eligible to take part in the study if they worked in Title X-funded settings and offered vasectomy services either in their own clinics or via referral. The sample was not intended to be representative but to include providers willing to discuss vasectomy perspectives and trends. Some of the participants were reluctant to talk about reproductive health services, given the politicized nature of the topic, especially in states with abortion bans. Thus, we do not provide information about our sample in congregate and do not offer details about provider specifics. The final project sample included 23 different organizations. Interviewees included physicians, nurse practitioners, nurses, mid-wives, and administrators. Interviewees represented state and local health departments, Planned Parenthood affiliates, non-profits, and federally qualified health centers. All organizations represented served low-income or under/uninsured individuals. Participants came from Title X entities in both rural and urban service areas and from all 10 HHS regions in all areas of the U.S. (e.g., Northeast, South, Midsouth, etc.). Participants also represented both abortion-restricted (e.g., abortion bans or gestational limits for abortion) and abortion non-restricted states.

### Procedures

Project staff interviewed participants one-on-one or in small groups of one to three interviewees depending on participant availability and preference. This study was approved by the second author's University IRB and all participants signed consents before being interviewed. The research team drafted an interview guide to meet project goals (e.g., gain understanding of existing vasectomy services and perspectives on those services) using existing concepts in the research and practice literature. A small group of collaborating researchers and reproductive health professionals reviewed and helped to revise the interview guide to create the final instrument. Questions centered on experiences offering vasectomy services. Example questions include: Describe the vasectomy services provided by your clinic; Explain how providers are trained to conduct vasectomies; Who seeks vasectomy services at your clinic? What barriers to clients face to accessing vasectomy services at your clinic? What changes or trends have you noticed in vasectomy over the last few years? What do you believe accounts for those changes? 23 different providers were interviewed in 21 interviews. Interviews took place on Zoom and lasted up to 1 h. Each interview was transcribed for analysis. Participants gave informed consent and received a $150 incentive for taking part in the interviews.

### Analysis

The team conducted a thematic analysis through an iterative process (22) with the aim of assessing the cultural, gender, and political perspectives on vasectomy services in Title X clinics. First, the analysis team read the transcripts and independently generated a list of key initial general themes describing vasectomy services. Next, the team met to compare and discuss their lists. In this process we expanded, consolidated, and redefined our initial ideas, and then created a more targeted master list of themes that we defined in a codebook for further exploration in the data. Then, the first three authors analyzed the same three transcripts independently using the codebook and met to discuss coding confusion or discrepancies. As a result, the team refined several code definitions and then proceeded to attach text to codes via the revised codebook to the remainder of the data. To ensure trustworthiness of coding between authors, in addition to engaging in test coding sessions to resolve discrepancies, the team also met regularly throughout the coding process to debrief and share coding questions and assure consistent application of the codebook. Throughout the analysis process the coders also wrote and discussed memos to note similarities across codes and interviews. Finally, we created a coding report which summarized themes, codes, and text and used this report to write the results section.

## Results

In this study, we sought perspectives from Title X providers who provide vasectomy services to help understand the cultural, gender, and political factors that may serve as barriers to access vasectomy services. Our qualitative interviews yielded key themes related to access, client culture, gender roles and expectations, language, and concern associated with growing concerns on reproductive health access. It is important to note that these findings are based on the perspective of providers, not individual clients. However, as they are working on the frontlines of publicly funded contraception services, their understanding of client experiences are based on their real-life experiences with their clients. And, their assumptions and perspectives are relevant, in a similar way that patients' are, to understanding what myths and challenges need to be addressed to better support vasectomy services.

### Culture: provider perspectives on sociocultural influences on vasectomy

Interviewees generally dismissed the idea that vasectomy was only of interest to specific groups of people. They talked mostly from a space of, “if you build it, they will come.” They noted that if you make vasectomies available, “people do it” and described their clinic's vasectomy clients as “a big mix,” noting that “we see [people] from ‘a wide range of different cultures.’ All interviewees seemed to believe knowing about vasectomies was important, however, and called for better systems to collect and manage data about their clients. In addition to conveying the opinion that vasectomies are for everyone and that assumptions otherwise could be unhelpful; interviewees did identify several sociocultural trends.”

#### Low-income communities face myriad social challenges

Respondents noted that in their experience, vasectomies were less accessible for low-income clients. Sometimes, this was a matter of being un and underinsured. In those cases, clinics with sliding scales discussed the benefits of financial assistance to help clients afford the procedure and noted the barriers that come with living in poverty. For instance, one respondent said that they saw that low paying (and often less flexible) jobs were problematic among their clinic population, especially if the procedure required a long wait or more than one appointment. Providers said this was similarly true of jobs in which clients also worried about the procedure's impact on them and their health beyond one day.

“[Clients say] that a big barrier is taking off work, especially if you're in a physical job where you have to take off … more [time] because you're doing construction.”

Several interviewees labeled transportation as a barrier for low-income clients at their clinics. A southern state Title X sub-recipient gave an example of a client who was going to have to drive four hours to get the vasectomy, which was a significant burden on time and funds. She also noted that clients often say they must weigh the cost of travel with the cost of the procedure:

“We try to work with other places in [the area] to … have other options for our patients … that don't want to travel as far, but the cost is usually the barrier there because [other providers] cost may not be as cheap as ours.”

Sometimes limited income forces people into difficult decisions. Interviewees noted that clients say that they do not always have enough discretionary funds to meet all their needs and give example like being forced to “decide if you're going to pay two months, rent or get a vasectomy.” One southern state grantee elaborated on another aspect of living on a limited income—what she perceived as emotional and public financial support gains that come with having children for vulnerable families. She said that what she experienced and heard from working with clients was that “Lower income persons may feel that's the one thing [a child] that [a man] can give a woman.” Such gains are only short-term, however, and do not reflect that families have informed and free choices. Instead, the provider was trying to indicate the many challenges of a low economic status. It is important nonetheless, that providers have these conversations, and in our interviews, they identified that low income and poor health environments can really shape how families think about having children and using contraception.

#### Providers note that language barriers hinder services

Another theme across the interviews was the barrier of language. Respondents noted the demographics of many of their clinics seemed to be changing and they served increasingly diverse populations who spoke languages other than English. This required clinics to rethink staffing and translation services. If clients could not fully understand the procedure and the coordination of scheduling the procedure, they were less likely to undergo a vasectomy. A West Coast community health center provider summarized this situation:

“We struggle with language barriers. For example, we have an increasing Hispanic community. We noticed this increase during COVID, and it keeps increasing. So sometimes the staff is not prepared for the changes, and [we hear from patients that] it’s hard for these patients to access those services if they don't have the interpretation services available. I know we have a lot of different translation phone systems, but sometimes, the translation is not accurate enough, and information gets lost in translation. [Then] patients have a second thought. They hear first this information, and now they go to the urologist, with another translation system, and [tell us] the translation is totally different.”

A mountain state FQHC reported that they did focus groups specifically to understand the needs of immigrants and others for whom English was not their first language. In doing so they produced information to appropriately reach and support these populations.

#### Providers worry that communities of color hold distrust

Interviewees noted several challenges that people of color express regarding vasectomies. These fears, of course, exist in the context of truly harmful and unfair treatment of communities of color by people in medicine and medical research (23). For one, they said that based on their experiences, they hear people of color express a lot of fear and uncertainty towards vasectomies. A provider from an FQHC in the northeast noted that she had conversations that revealed that the finality of vasectomies and the negative connotations of the finality of the procedure can be difficult for both staff *and* clients of color, particularly, to process. She said patients said they worried about making decisions that they could not change, especially if doctors were not giving them all of the information or enough of the correct information that they needed to make the decision. She said this led to misinformation and based on her patient experiences, that “there is some unlearning to be done in communities of color” and “[clinics] still have work yet to do” to address this, particularly regarding education, marketing, and communication. Another provider from a Midwest FQHC described a different aspect of this challenge. They said that in their experience, men of color do report not having as many community resources or support regarding vasectomy and lack places to go with their questions.

“If you are a man of color, you're not going to turn to your peers and hear that two of them have had vasectomies, and that it was no big deal the way white men do. It takes a lot of talking for some of them even when their wives are dragging them to their appointment.”

A west-coast Planned Parenthood affiliate noted, similarly, that the problem was somewhat cyclical. For instance, she said she believed that “unfortunately for brown and Black men, everyone's been told [they do not want vasectomies] for so long, that they [start to believe that they] don't want it. This was her impression only but speaks to her perspective based on treating men of color—which is important when considering the roles that patients and providers play in spreading correct medical information about vasectomies.”

### Gender: norms around women's roles in contraception

Interviewees noted that, socially, based on conversations they hear among patients, that contraception is assumed to be a woman's (and by default not a man's) responsibility. A provider from an Appalachian state said that although the health department had an area labeled family planning, that their perspective from their patient visits was that the general “understanding [among my clients is] that family planning is a female issue and not a male issue. You just don't have a lot of men who really think about it.” In talking about services in their region, A West Coast Planned Parenthood affiliate provider indicated that she frequently heard her patients refer to their service as “planned motherhood” and not “planned parenthood.” Another west coast Planned Parenthood provider summarized the issue well when she said –

“I think everybody, and I mean the society in general, sees reproductive health, parenting, pregnancies, as a woman’s thing. Men’s services get lost in the big picture. Even when you talk to men they will say, ‘birth control is a woman’s thing,’ it’s a woman’s responsibility.”

She also noted the irony of this in the current reproductive health environment, “That although we label reproductive health as a woman's thing, we are also currently taking away a woman's right to choose whether to continue a pregnancy.”

Interviewees described, from their clinic experiences, men's fear that vasectomies strip them of their masculinity. One respondent from a Mountain State community health center identified her service area as rural and conservative. She said that in her clinic she has heard men discuss that “men don't want to get a vasectomy because they feel like other men would make fun of them or they're getting neutered, that it makes you less of a man.” Another from a southern state grantee agreed with this and added that from what they experienced in conversations with their patients, men believed that their sexual abilities could decrease from a vasectomy.

A few participants also noted that from what they observe, some men use their ability to provide children as a control tactic and some said that some low-income men believe that they have “sperm in lieu of money.” One group participant said that she also experienced, via patient visits, that some men try to use children as a tactic in abusive relationships—and threaten that they won't “father any more of children, if [a partner] won't do this or that” Similarly, another participant elaborated that women told her stories about men holding children—or the possibility of having children “over their head” to exert power and control in the relationship. For example, one woman told her that her partner said that he would not support her financially if she did not have unprotected sex with him.

#### Partner assumptions

On the other hand, some interviewees said that they heard men say that did want to “do their part” but were inhibited by the perceptions of their female partners. A west coast health department employee said that often they have more contact with the women in the clinic, but they hear women making comments that assume their partners would “never” be willing to get a vasectomy. She did not believe that assumption was necessarily true; however, another said this was important, because based on their experience, they believed that men often “have to be convinced to get a vasectomy from their partner.” They cautioned, though, that since many people lack information about vasectomies, it is hard for women to be held responsible for educating men about the procedure and that they may not have adequate information to discuss vasectomies accurately. In this way they believed, based on what they witnessed among patients, that myths about vasectomies were perpetuated, such as it that the procedure could be “painful” or “could make someone less of a man” or could alter ejaculation.

#### Missed opportunities to reach men

Interviewees also discussed other provider's assumptions or lack of information about how to reach and treat men. One Planned Parenthood provider noted that many laboratory nurses at her facility had very few experiences treating men. She explained an experience when they did not know what to do with post vasectomy semen analyses. Another said that men often come in for other varied reasons than reproductive health, such as an STI concern, but that she sees too few providers using these opportunities to offer or ask about other services, because this is how she has found out that men may be interested in vasectomies. She said that they started a patient navigation program where the patient navigators ask patients post appointment follow-up questions. So even if they only see the female partner, they ask—“Do you think that your partner would like to set up an appointment for a consult and see if we can refer him to get the services?” One representative from a rural health department noted that they were in the habit of asking every woman if she was planning to get pregnant but said that she and her colleagues did not also ask this of men.

“What I wanted to do was pilot asking every man at their wellness visits, ‘Are you intending to get someone pregnant within the next year?’ And then the follow-up question is, well, if not, what are you doing to prevent that?” That's the standard of practice for seeing women in reproductive health, but not necessarily men. I think that could be a path forward because it opens the door to education and allows providers to have this conversation with their patients who can impregnate someone else, and it also gives those patients the message that ‘Oh, contraception is for me, too. This isn't just something for my female partner. This is something I can also think about.’ The medical world doesn't always convey that.”

Another interviewee added that care providers “could do better,” especially with the political, cultural climate, normalizing vasectomy and talking about vasectomy as a great option.

### Political: men step up

Despite many of the barriers noted, interviewees also discussed that they noticed slight differences and trends around some men “stepping up” given the current reproductive health climate. One interviewee said that she experienced, through conversations with patients, men's increased interest in vasectomies—“as soon as Roe was overturned, everyone got real worried, what are we going to do? And men … stepped up to the plate.” Another noted that clients are “not as shy” to ask about vasectomies, and this has prompted them to present more men's health information at their health education events. A West Coast Planned Parenthood affiliate respondent said she noticed “political factors” coming into play with “young men” specifically after the Dobbs decision—that some men said that they “pursued the procedure after [Dobbs] specifically.” Another Midwest Planned Parenthood respondent agreed that “I've gotten more calls and more people stating that they were worried about their [female] partners [given the overturn of Roe v. Wade].”

#### Panic and increased demand

Interviewees also commented on the lack of longer-term reproductive methods in general given the current political climate, noting that people called in a panic and said that they “never wanted to worry about access again.”

“Anytime that there is a political threat to access to family planning services that we see an uptick in both women using long-acting reversible methods and also men requesting vasectomy services.”

Another said that clients raised “the post Dobbs” climate multiple times with her, and she noticed the increased volume of vasectomy services. She said they used to do about one vasectomy a month, but now they were doing “two or three a week with 20 [more] booked on the schedule.” An urban FQHC in the Northeastern US said that their clinic's vasectomies have tripled, and some clients were concerned about access and waiting times. Most others agreed that demand needed to be met with increased support and ability.

## Discussion

This study revealed a host of sociocultural, gender, and politically based factors influencing the provision of and access to vasectomy services in Title X funded health centers in the U.S. The analysis reveals four broad categories of strategies that Title X grantees and health centers can consider when addressing these challenges.

### Messaging and education

Our analysis identified several challenges associated with client awareness, understanding, and misconceptions about vasectomy. Creating messages, materials, and communication tools to educate both those receiving the vasectomy and their partners can go a long way in dispelling myths and increasing interest in vasectomies. It may also be necessary to develop targeted messaging for vulnerable sub-groups to address specific cultural or gendered concerns. When possible, offering materials and counseling/education in a variety of languages will also support messaging for a diverse audience of clients. Evaluated public health campaigns focused on vasectomy are lacking and present a future needed focus area of practice and research. One study found that men talk to each other about the procedure and that these interpersonal conversations could be harnessed for health education campaigns (23).

### Addressing mistrust

Many in the BIPOC community have a deep-rooted mistrust of permanent contraception based on very real historical and present injustices like discrimination (24). Less research focuses on men's experiences, but our findings indicate that the provider views in our study match those expressed by men in the research that does exist. Ngyuen et al. (25) found, for example, that Black men's use of male focused contraception was limited by medical mistrust (of doctors, pharmaceutical companies, medicines). Participants referenced specific concerns. As one man put it, “Are you really trying to help us with birth control? Are you trying to kill my people off […] Are you trying to deform my people? Are you trying to stop us from reproduction?”

Health care providers exhibit the same amounts of implicit bias as the general population—which can affect their medical evaluations and judgements (26). It is crucial that all health center staff are trained in understanding those historical roots of mistrust and in how to minimize implicit bias. Many training programs exist and some states even mandate these learning opportunities as a solution (27). Successful trainings include opportunities for learners to use modules and take advantage of opportunities to interact with diverse groups of patients (28).

Individual-level provider biases are only one level of the problem. Structural level challenges need to be addressed to help eliminate health disparities (29). These include broad challenges like poverty, lack of access to education and care, as well as structural challenges in medicine itself. Providers may not sufficiently learn about mistrust and discrimination in their curricula, and they may lack educators and role models who demonstrate patient-centered care (30), suggesting that more attention be paid to who teaches and mentors medical students. Similarly, overall inequities in access to higher education contribute to lower numbers of diverse medical providers (28). Once practicing, providers may lack support to adequately consider how to support patient decisions. For example, a study of emergency room doctors indicated that stress driven by high patient load was associated with higher levels of racial bias, suggesting that decreasing the burden of care in these settings could limit bias and improve patient-centered care and attention (31).

### Clinical practices to address socio-cultural barriers to accessing vasectomy services

Our study identified a host of challenges associated with employment, geographic distance, and transportation. For many lower income clients, the ability to take time off from their jobs poses a significant challenge. In some states, clients must travel great distances for the appointments and procedure, adding additional time off and cost implications. The traditional three-visit model [Consultation, Procedure, Post Vasectomy Semen Analysis (PVSA)] further compounds these barriers. Title X health centers can adopt practices that simplify these three steps to accommodate for issues like jobs, cost, transportation, and distance. Some providers said that they found success in conducting the initial consultation via telehealth. Others used their network of providers to offer the initial consultation at multiple locations, minimizing the need to travel long distances for that visit. There are others who have taken advantage of options for mailing in a sample for the PVSA. All of these strategies are recommended to make vasectomy more accessible for people with low incomes and multiple life demands.

### Sexual and reproductive health services for all

Finally, as many of the interviewees noted, the Dobbs decision had a significant impact on reproductive autonomy for women, a trend found in other emerging research (32). Title X grantees and service sites must ensure that they are offering comprehensive contraceptive services for women, men, transgender, and non-binary individuals to maintain and improve SRHW (6). Creating and training staff to use protocols and screening that also address the reproductive intentions of men and sperm-producing clients is a first step toward opening the dialogue and normalizing the conversation around vasectomy (6, 23, 25).

## Limitations

This study faces a few limitations. First, the findings are not based on a representative sample of Title X organizations and there is limited representation from a range of states in the southern United States. The southern states consistently have some of the poorest reproductive health outcomes and includes multiple states with abortion bans and/or restrictions. Additionally, these findings are based on the perspectives, assumptions, and opinions of providers, not clients themselves. We are working on the assumption that as frontlines workers in publicly funded SRH organizations, they bring powerful perspectives based on their daily interactions. Nonetheless, it is possible that their personal perspectives reflect their own assumptions and biases. Additional research on patient perspectives is important.

## Data Availability

The raw data supporting the conclusions of this article will be made available by the authors, without undue reservation.

## Footnotes

^1^A Note on Gender: It is important to note that not all clients seeking vasectomy services will identify as male and not all will have partners that identify as female. However, since this manuscript focuses on both cultural and gender influences, we have used the use the terms “man” or “male” for sperm-producing clients and “woman” or “female” for people with uteruses.

## References

[1] Centers for Disease Control and Prevention (CDC). Reproductive Health 2023 [updated March 27, 2023].

[2] Hart J Crear-Perry J Stern L. US sexual and reproductive health policy: which frameworks are needed now, and next steps forward. Am J Public Health. (2022) 112(S5):S518–s22. 10.2105/AJPH.2022.306929 35767777 PMC10490305

[3] Gomez AM Bennett AH Arcara J Stern L Bardwell J Cadena D Estimates of use of preferred contraceptive method in the United States: a population-based study. Lancet Reg Health Am. (2024) 30:100662. 10.1016/j.lana.2023.100662 38304390 PMC10831268

[4] Hamm M Evans M Miller E Browne M Bell D Borrero S. “It’s her body”: low-income men’s perceptions of limited reproductive agency. Contraception. (2019) 99(2):111–7. 10.1016/j.contraception.2018.10.005 30336131 PMC6744607

[5] Plana O. Male contraception: research, new methods, and implications for marginalized populations. Am J Mens Health. (2017) 11(4):1182–9. 10.1177/1557988315596361 26206159 PMC5675331

[6] Witte C Yost F Suennen L Craven M. US sexual and reproductive health policy: which frameworks are needed now, and next steps forward (2024). Available online at: https://www.asktia.com/article/dear-future-president-what-women-want-for-healthcare-in-america (accessed August 01, 2024). 10.2105/AJPH.2022.306929 PMC10490305 35767777

[7] Centers for Disease Control and Prevention (CDC). Male Sterilization 2023 [updated March 27, 2023].

[8] Daniels K Abma JC. Current Contraceptive status among Women Aged 15–49: United States, 2017–2019. Hyattsville, MD: National Center for Health Statistics (2020). 33151146

[9] Borrero S Moore CG Creinin MD Ibrahim SA. Low rates of vasectomy among minorities: a result of differential receipt of counseling? Am J Mens Health. (2010) 4(3):243–9. 10.1177/1557988309337619 19706674 PMC2891806

[10] Sellke N Tay K Sun HH Tatem A Loeb A Thirumavalavan N. The unprecedented increase in Google searches for “vasectomy” after the reversal of roe vs. wade. Fertil Steril. (2022) 118(6):1186–8. 10.1016/j.fertnstert.2022.08.859 36180257

[11] Alef R Mayrovitz HN. Assessing current interest, knowledge, and inquiries about vasectomies in urology patients and medical students. Cureus. (2023) 15(10):e47074. 10.7759/cureus.47074 38021500 PMC10644118

[12] Bole R Lundy SD Pei E Bajic P Parekh N Vij SC. Rising vasectomy volume following reversal of federal protections for abortion rights in the United States. Int J Impot Res. (2023) 36:265–8. 10.1038/s41443-023-00672-x. 36788351 PMC9925925

[13] Sax MR. Seeking vasectomy in post-Dobbs America: the male counterpart response to the reversal of roe v wade as evidenced by Google search trends. Fertil Steril. (2022) 118(6):1189. 10.1016/j.fertnstert.2022.10.006 36369185

[14] Nguyen BT. The demand for male contraception: estimating the potential market for users of novel male contraceptive methods using United States national survey of family growth data. Contraception. (2024) 135:110438. 10.1016/j.contraception.2024.110438 38555051

[15] Datta PK Chowdhury SR Aravindan A Nath S Sen P. Looking for a silver lining to the dark cloud: a google trends analysis of contraceptive interest in the United States post roe vs. wade verdict. Cureus. (2022) 14(7):e27012. 10.7759/cureus.27012 35989835 PMC9386304

[16] Ranji U Gomez I Salganicoff A Rosenzweig C Kellenberg R Gifford K. Medicaid coverage of family planning benefits: findings from a 2021 state survey. Kaiser Family Foundation (2022).

[17] White K Martínez Órdenes M Turok DK Gipson JD Borrero S. Vasectomy knowledge and interest among U.S. men who do not intend to have more children. Am J Mens Health. (2022) 16(3):15579883221098574. 10.1177/15579883221098574 35562856 PMC9112422

[18] Mortach S Sellke N Rhodes S Sun HH Tay K Abou Ghayda R Uncovering the interhospital price variations for vasectomies in the United States. Int J Impot Res. (2024). 10.1038/s41443-024-00833-6 38383856

[19] Brown BP Chor J. Adding injury to injury: ethical implications of the medicaid sterilization consent regulations. Obstet Gynecol. (2014) 123(6):1348–51. 10.1097/AOG.0000000000000265 24807338

[20] Borrero S Zite N Potter JE Trussell J. Medicaid policy on sterilization–anachronistic or still relevant? N Engl J Med. (2014) 370(2):102–4. 10.1056/NEJMp1313325 24401047 PMC4418554

[21] Thiel de Bocanegra H Cross Riedel J Menz M Darney PD Brindis CD. Onsite provision of specialized contraceptive services: does title X funding enhance access? J Womens Health (Larchmt). (2014) 23(5):428–33. 10.1089/jwh.2013.4511 24405313 PMC4011460

[22] Guest G MacQueen KM Namey EE. Applied Thematic Analysis. Thousand Oaks: Sage Publications (2012).

[23] White AL Davis RE Billings DL Mann ES. Men’s vasectomy knowledge, attitudes, and information-seeking behaviors in the southern United States: results from an exploratory survey. Am J Mens Health. (2020) 14(4):1557988320949368. 10.1177/1557988320949368 32812507 PMC7444157

[24] Bazargan M Cobb S Assari S. Discrimination and medical mistrust in a racially and ethnically diverse sample of California adults. Ann Fam Med. (2021) 19(1):4–15. 10.1370/afm.2632 33431385 PMC7800756

[25] Nguyen BT Brown AL Jones F Jones L Withers M Ciesielski KM “I'm not going to be a Guinea pig:” medical mistrust as a barrier to male contraception for black American men in Los Angeles, CA. Contraception. (2021) 104(4):361–6. 10.1016/j.contraception.2021.06.001 34118271 PMC8857976

[26] FitzGerald C Hurst S. Implicit bias in healthcare professionals: a systematic review. BMC Med Ethics. (2017) 18(1):19. 10.1186/s12910-017-0179-8 28249596 PMC5333436

[27] Cooper LA Saha S van Ryn M. Mandated implicit bias training for health professionals—a step toward equity in health care. JAMA Health Forum. (2022) 3(8):e223250. 10.1001/jamahealthforum.2022.3250 36218984

[28] Lett E Orji WU Sebro R. Declining racial and ethnic representation in clinical academic medicine: a longitudinal study of 16 US medical specialties. PLoS One. (2018) 13(11):e0207274. 10.1371/journal.pone.0207274 30444928 PMC6239326

[29] Vela MB Erondu AI Smith NA Peek ME Woodruff JN Chin MH. Eliminating explicit and implicit biases in health care: evidence and research needs. Annu Rev Public Health. (2022) 43:477–501. 10.1146/annurev-publhealth-052620-103528 35020445 PMC9172268

[30] Jochemsen-van der Leeuw HG van Dijk N van Etten-Jamaludin FS Wieringa-de Waard M. The attributes of the clinical trainer as a role model: a systematic review. Acad Med. (2013) 88(1):26–34. 10.1097/ACM.0b013e318276d070 23165277

[31] Johnson TJ Hickey RW Switzer GE Miller E Winger DG Nguyen M The impact of cognitive stressors in the emergency department on physician implicit racial bias. Acad Emerg Med. (2016) 23(3):297–305. 10.1111/acem.12901 26763939 PMC5020698

[32] Adler A Biggs MA Kaller S Schroeder R Ralph L. Changes in the frequency and type of barriers to reproductive health care between 2017 and 2021. JAMA Netw Open. (2023) 6(4):e237461. 10.1001/jamanetworkopen.2023.7461 37036704 PMC10087056

